# Application of emerging information technologies in the prevention and control of chronic diseases

**DOI:** 10.3389/fpubh.2026.1755672

**Published:** 2026-01-21

**Authors:** Tong Feng, Yi Li, Yinhe Feng, Chunfang Zeng

**Affiliations:** Department of Respiratory and Critical Care Medicine, Deyang People's Hospital, Deyang, China

**Keywords:** artificial intelligence, chronic non-communicable diseases, digital health technologies, internet plus, wearable devices

## Abstract

Chronic non-communicable diseases (NCDs)—including cardiovascular disease, diabetes, chronic obstructive pulmonary disease (COPD), and chronic kidney disease—pose a major 21st-century global public health challenge. They drive high morbidity, mortality, and escalating healthcare costs. Traditional reactive, clinic-centered care models are ill-equipped to meet the ongoing, complex needs of chronic disease patients. This has prompted a shift toward proactive, personalized, and patient-centered approaches. This narrative review examines the transformative potential of emerging digital health technologies (DHTs) in chronic disease prevention and control. It emphasizes the synergistic integration of four key domains: Internet Plus ecosystems, wearable devices and sensors, artificial intelligence (AI) and machine learning, and interactive voice-based follow-up or conversational agents. Internet Plus serves as the foundational infrastructure. It enables seamless data integration, care coordination, telemedicine, and patient empowerment across stakeholders. Wearable devices facilitate continuous, real-time monitoring of physiological and behavioral data, yielding valuable insights for timely interventions in cardiovascular, metabolic, respiratory, and musculoskeletal disorders. AI and machine learning drive predictive diagnostics, risk stratification, and personalized digital therapeutics, demonstrating superior efficacy and cost-effectiveness in areas like pulmonary rehabilitation and orthopedic care. Voice-based technologies provide scalable, low-cost solutions for medication adherence, symptom monitoring, and health education. They particularly benefit older adults and rural populations. Despite these advances, significant challenges remain. These include data security and privacy risks, health inequities amplified by the digital divide and device biases, and AI limitations (e.g., reproducibility, opacity or “black-box” issues, and unclear legal accountability). In conclusion, the convergence of these technologies promises a more precise, proactive, and inclusive paradigm for chronic disease management. Future success hinges on robust privacy protections, inclusive design, diverse real-world validation, and refined regulatory frameworks to ensure equitable and sustainable implementation.

## 1 Introduction

Conditions such as cardiovascular disease, diabetes, chronic obstructive pulmonary disease (COPD), and chronic kidney disease (CKD) are leading causes of morbidity, mortality, and rising healthcare costs worldwide (1). Traditional healthcare models—often characterized by episodic, reactive care in clinical settings—are increasingly strained by the complex, continuous needs of chronic disease patients. This mismatch between the nature of chronic disease and the structure of care delivery has catalyzed a fundamental shift toward more proactive, personalized, and patient-centered approaches.

Digital health technologies (DHTs) have emerged as a transformative force in this landscape, offering innovative tools to bridge critical gaps in chronic disease prevention and control. The integration of four key technological domains—Internet Plus ecosystems, wearable devices and sensors, artificial intelligence (AI) and machine learning (ML), and voice-follow-up/conversational agents—creates a synergistic framework capable of supporting continuous monitoring, data-driven insights, and sustained patient engagement (2, 3). The Internet Plus model, which integrates the internet with traditional industries, provides the foundational infrastructure for connecting disparate elements of the healthcare ecosystem, from patients at home to clinicians in hospitals.

**Figure 1:**
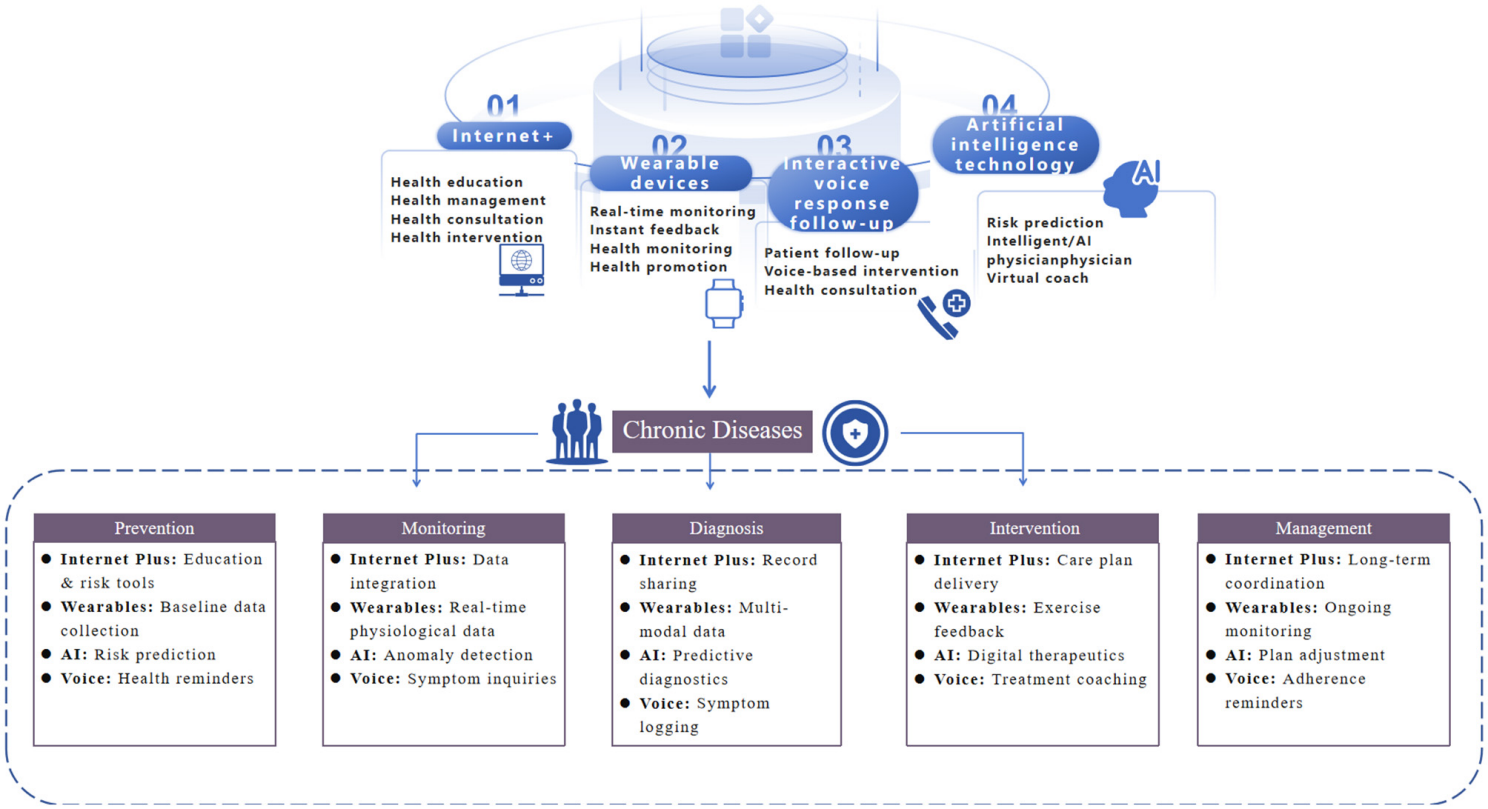
Applications of emerging information technologies in chronic disease prevention and control.

This review synthesizes current evidence and conceptual frameworks to explore how the convergence of these four digital health technologies is reshaping chronic disease prevention and control. It examines the role of Internet Plus as an enabling ecosystem, the capabilities of wearables for continuous monitoring, the analytical power of AI/ML for generating insights, and the engagement potential of conversational agents. The discussion also critically addresses implementation barriers, equity concerns, and future directions for creating effective, inclusive, and scalable digital health solutions for the global chronic disease burden Figure 1.

## 2 Literature search and selection methods

This article is a narrative review aimed at summarizing representative applications, achievements, and challenges of emerging information technologies in chronic disease prevention and control, rather than a systematic review or meta-analysis. We comprehensively searched English-language literature in the PubMed and Web of Science databases. The search was restricted to title/abstract fields using combinations of the following key terms: (“chronic disease” OR “non-communicable diseases” OR “NCDs”) AND (“intervention” OR “prevention” OR “management” OR “health care” OR “health education”) AND (“internet” OR “Internet+” OR “wearable device” OR “interactive voice response” OR “artificial intelligence”). The search covered publications from January 1, 2020, to November 1, 2025, to focus on recent advances aligned with the rapid development of these technologies.

We prioritized high-quality studies, including randomized controlled trials (RCTs), large-scale cohort studies, meta-analyses, and authoritative reviews that demonstrated clear clinical or public health significance. No formal quality assessment tools (e.g., PRISMA checklist) were applied, as the objective was to synthesize insightful and representative evidence rather than to exhaustively include all eligible studies. References cited in identified articles were also manually screened to supplement key literature.

## 3 Four emerging information technologies in chronic disease prevention and control

### 3.1 Internet plus as a foundational ecosystem for integrated chronic disease management

The “Internet Plus” concept, which signifies the deep integration of internet technology with traditional sectors, provides the essential connective tissue for a modern, integrated chronic disease management system. In healthcare, this translates to a digital ecosystem that seamlessly links patients, caregivers, primary care providers, specialists, and health administrators, facilitating data flow, communication, and coordinated care across traditional boundaries of time and space. This ecosystem is not a single application but a networked platform that enables the other three technological pillars—wearables, AI, and conversational agents—to function in a unified, scalable manner.

At its core, the Internet Plus ecosystem enables Comprehensive Data Integration and Sharing. Chronic disease management generates data from a myriad of sources: continuous glucose monitors, Bluetooth-enabled blood pressure cuffs, smartphone apps logging diet and mood, EHRs from clinic visits, and even environmental sensors. An Internet Plus platform, such as China's proposed integration of AI tools such as China's exploration of open-source large language models like DeepSeek for integration into primary care settings or Brunei's national BruHealth platform, acts as a central hub that aggregates this information (4, 5). This breaks down data silos, allowing for a holistic view of the patient. For instance, the ADLIFE digital intervention for advanced chronic disease integrated individualized care plans and decision support across pilot sites in Europe, demonstrating how a shared platform can standardize and improve care coordination across different health systems (1).

This integrated data foundation directly supports Enhanced Care Coordination and Telemedicine. The platform enables asynchronous and synchronous communication between all stakeholders. Healthcare providers can remotely monitor patient-generated data, identify trends indicating deterioration, and intervene proactively through virtual consultations or adjusted care plans. This model is particularly crucial for managing multimorbidity, where patients interact with multiple specialists. The ProACT platform, for example, is designed to support older adults with multimorbidity by connecting them with a formal and informal care network via a digital health platform, aiming to improve care coordination and self-management support. Similarly, Internet-based proactive follow-up models for conditions like non-alcoholic fatty liver disease (NAFLD) have been shown to significantly improve patient compliance and quality of life compared to traditional telephone follow-up (6).

Furthermore, the ecosystem empowers Patient Empowerment and Self-Management. Through patient portals and mobile applications tethered to the central platform, individuals gain access to their health data, educational resources, and personalized feedback. This transparency and engagement are critical for fostering self-efficacy. Studies on digital interventions for medication safety in adults with diabetes and for type 2 diabetes mellitus (T2DM) self-management in Saudi Arabia highlight the importance of accessible, theory-informed digital tools that patients can use in their daily lives to manage complex regimens (7). The platform can deliver structured programs, such as the 16-week BALANCE program for diabetes management, which combines education, personalized plans, and health coaching within a national app ecosystem (4).

### 3.2 Wearable devices and sensor technologies: enabling continuous monitoring and data generation

Wearable devices and embedded sensor technologies represent the frontline data-gathering layer in the digital health ecosystem, transforming sporadic clinical measurements into continuous, real-time streams of physiological and behavioral information. This shift from episodic to continuous monitoring is fundamental for chronic disease management, as it captures the dynamic nature of conditions like hypertension, diabetes, and COPD within the context of an individual's daily life. The data generated by these devices provide the raw material for AI-driven insights and form the basis for personalized feedback and timely clinical interventions.

The Clinical Applications and Impact of wearables are vast and growing. In cardiovascular and metabolic disease management, devices such as continuous glucose monitors (CGMs), smart blood pressure cuffs, and heart rate monitors are now well-established. Studies demonstrate that integrating real-time CGM data with personalized digital health coaching leads to significant short-term improvements in glycemic control and lifestyle behaviors in individuals with type 2 diabetes and prediabetes, particularly among those with high glycemic variability (8). Remote patient monitoring (RPM) programs that provide patients with Bluetooth-enabled BP cuffs and link data to clinical teams have been associated with clinically meaningful reductions in systolic blood pressure, even in complex patients with multiple chronic conditions (9).

In respiratory and musculoskeletal disorders, wearables offer novel capabilities for objective assessment and rehabilitation. For COPD, chest-worn devices like the Respeck patch can derive continuous breathing waveforms, from which features like respiratory rate variability can be extracted. These objective metrics show correlation with subjective symptom burden scores like the COPD Assessment Test (CAT), offering a potential tool for remote exacerbation detection (10). In musculoskeletal digital therapeutics, wearable sensors enable AI-driven motion analysis for personalized rehabilitation programs, improving adherence and functional outcomes (11).

The evolution of technology is driving a trend toward Multi-Modal and Clinical-Grade Wearables. Early consumer fitness trackers are giving way to more sophisticated medical devices capable of capturing multiple physiological signals simultaneously. The Perin Health Patch (PHP), for example, integrates six sensing modalities—auscultation, electrocardiography (ECG), photoplethysmography (PPG), bioimpedance (BioZ), skin temperature, and motion—into a single chest-worn device (12). This convergence allows for the calculation of advanced metrics, such as the pulse respiration quotient (PRQ) from BioZ and ECG data, which can indicate cardiorespiratory resilience. Such multi-modal devices aim to replicate, in a wearable form, the combined functionality of several standalone clinical tools like Holter monitors, spirometers, and pulse oximeters.

### 3.3 Artificial intelligence and digital therapeutics: from predictive diagnosis to personalized intervention

Artificial intelligence represents the cognitive core of the digital health revolution, offering unparalleled capabilities to analyze complex, high-dimensional data and generate insights for chronic disease care. AI's role extends across the entire care continuum, from early risk prediction and improved diagnosis to the development of personalized treatment plans and digital therapeutics (DTx)—software-based interventions regulated as medical devices. Machine learning algorithms, including random forests, gradient boosting machines, and deep neural networks, excel at identifying subtle patterns within clinical, imaging, genomic, and lifestyle data that may elude conventional analysis. This enables a shift from reactive, population-based protocols to proactive, individualized management strategies.

Predictive modeling using AI has shown significant promise for early risk stratification across various chronic diseases. For metabolic dysfunction-associated fatty liver disease (MAFLD), a novel two-stage contrastive learning method that integrates clinical and survey-based data significantly outperformed traditional benchmarks in predicting disease phenotypes, supporting personalized care planning (13). In COPD, machine learning analysis of transcriptomic data identified endoplasmic reticulum stress-related genes like DNAJB1 as potential diagnostic biomarkers, offering new avenues for early detection (11). Similarly, for CKD, a portable electrical impedance tomography system combined with machine learning achieved 93% accuracy in classifying disease stages, presenting a non-invasive screening tool for community settings (14). These tools empower clinicians to intervene earlier, potentially altering disease trajectories.

Digital therapeutics, powered by AI, deliver structured, evidence-based interventions directly to patients. In chronic respiratory disease, DTx for pulmonary rehabilitation demonstrated not only superior efficacy to standard treatment in a randomized controlled trial but also cost-effectiveness, with an incremental cost-utility ratio well below willingness-to-pay thresholds (15). This provides a compelling value-based pricing framework for integration into healthcare systems. For musculoskeletal disorders, DTx and digital rehabilitation platforms report outcomes comparable or superior to traditional approaches, with adherence gains of 15%−40% and cost reductions of 30%−40%, highlighting their transformative potential in orthopedic care (16). These interventions often incorporate AI-driven motion analysis, personalized exercise regimens, and tele-rehabilitation support.

Personalized intervention is further advanced by AI's ability to integrate multi-omics data and provide explainable insights. Research on suboptimal health status used an elastic net model on transcriptomic, metabolomic, and microbiome data to achieve high prediction accuracy, with Shapley Additive exPlanations (SHAP) analysis revealing individual-level biological drivers for tailored preventive strategies (17). In dietetics, AI-integrated technologies like image recognition for food logging and chatbots for nutrition counseling are being explored to support personalized medical nutrition therapy, though concerns regarding ethics and patient safety remain (18). The emerging field of explainable AI (XAI) is crucial for clinical adoption, with methods like SHAP and LIME helping to demystify model decisions and build trust among healthcare professionals. As these technologies mature, they promise to usher in an era of precision chronic disease management where interventions are dynamically tailored to each individual's unique biological makeup, lifestyle, and real-time health data.

### 3.4 Remote follow-up and collaborative care: building a patient-centered chronic disease management ecosystem

With the ongoing aging of the population and the persistent rise in the incidence of chronic diseases (such as diabetes, cardiovascular diseases, and chronic obstructive pulmonary disease), traditional healthcare resources are facing tremendous pressure. Interactive intelligent voice technology—including Interactive Voice Response (IVR) systems, voice virtual assistants (e.g., Amazon Alexa and Google Assistant), and dedicated offline assistants (e.g., ACHO)—is emerging as a low-cost, scalable tool. It shows considerable potential for chronic disease prevention, control, and health follow-up. This technology enables medication reminders, symptom monitoring, health education, and follow-up management through voice interaction, making it particularly suitable for older adults and populations in rural areas, where it can effectively improve treatment adherence and self-management capabilities.

During the COVID-19 pandemic, voice assistants were employed for chronic disease follow-up and appointment management, enhancing patient satisfaction and adherence (19). A qualitative study explored the experiences and perspectives of primary care nurses in the Extremadura region of Spain regarding the use of the offline voice virtual assistant Assistant on Care and Health Offline (ACHO) to support treatment adherence in older adults with chronic conditions. Through in-depth interviews, the study found that nurses generally acknowledged the usability of ACHO, its offline functionality, and customizable features, believing that it contributed to promoting patient independent living, optimizing medical data collection, and strengthening follow-up management (20).

Interactive intelligent voice technology has demonstrated preliminary efficacy in health follow-up and chronic disease prevention and control, particularly in the areas of medication adherence, symptom monitoring, and home-based management, where it can complement traditional healthcare models and enable proactive health interventions (21).

## 4 Challenges and concerns

### 4.1 Data security and privacy protection

Health data constitute the foundational element for the application of emerging information technologies. Through the processing and analysis of large volumes of health data collected by healthcare professionals or medical devices, as well as user-generated data from multiple sources, it is possible to gain comprehensive insights into patients' health status and behavioral patterns, thereby enabling disease risk prediction, health management, and precision medicine. However, health data encompass substantial amounts of sensitive personal information, including genetic resources. Any breach or leakage of such data can severely impact patients' employment and daily life and raise serious ethical concerns.

Internationally, regulations such as the EU's General Data Protection Regulation (GDPR), the US Health Insurance Portability and Accountability Act (HIPAA), and China's Personal Information Protection Law (PIPL) provide frameworks for protecting health data privacy, emphasizing principles like data minimization, consent, and de-identification. In China, the PIPL classifies medical and health data as sensitive personal information requiring heightened protection, including explicit consent and impact assessments for processing.

Privacy-enhancing technologies (PETs), such as federated learning (enabling model training without centralizing data), differential privacy (adding noise to datasets to prevent re-identification), and homomorphic encryption, offer promising applications in health data management by allowing analysis while preserving privacy (22). However, challenges persist, including computational overhead, potential reductions in data utility/accuracy, and difficulties in full implementation across diverse systems. Chikwetu et al. have emphasized that merely eliminating direct personal identifiers does not adequately ensure privacy protection. They note that sophisticated malicious actors can utilize advanced techniques, including machine learning, to re-identify individuals, reconstruct the original datasets, or infer additional sensitive information not originally disclosed (23). Although advanced PETs have been developed, fully effective and comprehensive privacy protection solutions remain lacking.

Therefore, in the collection and utilization of health data, paramount emphasis must be placed on data security and privacy protection. This requires strengthened information security education, the establishment of robust regulatory and oversight mechanisms for information protection, and clear accountability systems. Only through these measures can a secure foundation be laid for the legitimate use and sharing of health data.

### 4.2 Health equity concerns

Although emerging chronic disease prevention and control measures grounded in internet-based technologies have improved efficiency and expanded the reach of beneficiaries, patients must possess at least a computer, smartphone, or smart wearable device to derive health benefits from these technological advancements. Furthermore, the majority of efficacy evaluations of these novel technologies have been conducted in high-income populations or younger individuals with greater acceptance of smart devices, resulting in limited generalizability of findings.

The “digital divide” is prominent across populations. Urban residents typically have better broadband infrastructure and device access than rural areas, where connectivity is limited. Older adults face barriers from lower digital literacy and physical limitations. Individuals with lower education or socioeconomic status are less likely to adopt or effectively use these digital tools for chronic disease management. For example, studies have shown racial biases in devices like pulse oximeters and lower accuracy of wearable atrial fibrillation detection in older adults cohorts. Ford et al. conducted an evaluation of the Apple Watch and its proprietary algorithm for detecting atrial fibrillation within an older adults' demographic, characterized by a mean participant age of 76 years. Their findings revealed a sensitivity of merely 19%, a figure substantially lower than the 96% sensitivity rate previously asserted by the manufacturer (24). Separately, a United States-based investigation into the accuracy of pulse oximeters across different racial groups demonstrated that Black patients had an approximately threefold higher likelihood than White patients of experiencing occult hypoxemia that went undetected by the devices (25).

To address these inequities, strategies such as “inclusive design” (e.g., user-centered approaches incorporating diverse community input, accessible interfaces, and multilingual support) and “inclusive digital health” initiatives (e.g., subsidized devices, community training programs, and offline alternatives) are essential. Extensive research is warranted to assess the real-world performance of these technologies across diverse populations, to investigate health inequities arising from disparities in device accessibility, and to develop targeted solutions.

### 4.3 Challenges in the Application of Artificial Intelligence

In recent years, an increasing number of technical interventions have incorporated AI technologies—including machine learning, artificial neural networks, natural language processing, and expert systems—to enhance performance and effectiveness. However, AI remains in a relatively immature stage of development, and supporting standards and regulatory frameworks are not yet fully established.

First, AI outputs are heavily influenced by underlying algorithms, initialization conditions, and variable configurations; when confronted with identical data or scenarios, models based on different algorithms often yield substantially divergent results, compromising reproducibility (26). Second, computational models relying on artificial neural networks contain “hidden layers” that render their decision-making processes opaque—a characteristic known as the “black-box” problem—making it difficult to ascertain the reasons behind specific outputs or to understand why certain processes fail. Transparency requirements, such as XAI) techniques, are critical in medical decision-making to build clinician trust and enable oversight (27).

Third, the legal status of AI entities remains ambiguous, and accountability for adverse outcomes is complex; when harm arises from AI-driven decisions, attribution of responsibility is contentious and requires broad discussion and clear definition (28). Frameworks like the EU AI Act and WHO Ethics and Governance of AI for Health guidelines emphasize shared accountability: developers for algorithmic flaws and bias, medical institutions for deployment and oversight, and regulators for enforcement and standards. Therefore, alongside the advancement of AI technology, it is imperative to formulate application standards, improve relevant laws and regulations, and explicitly delineate liability for adverse consequences caused by AI, thereby facilitating its safe and widespread adoption.

## 5 Research trends and future directions

The true transformative potential of these technologies lies in their synergistic integration rather than isolated application. Internet Plus acts as the foundational ecosystem that enables seamless data flow and coordination among the other three pillars. Wearable devices generate continuous, real-time multimodal data that serve as the primary input for AI-driven predictive analytics and personalized interventions. AI, in turn, processes these data streams to produce actionable insights—such as early risk alerts or tailored digital therapeutics—which are then delivered back to patients via Internet Plus platforms. Voice-based conversational agents enhance patient engagement by providing accessible, natural-language interfaces for education, reminders, and symptom reporting, thereby improving adherence to AI-recommended plans and closing the feedback loop with new patient-generated data.

For example, in diabetes management, wearable continuous glucose monitors feed real-time data through an Internet Plus platform to AI algorithms for glycemic pattern recognition and predictive hypoglycemia alerts; voice agents then deliver timely reminders or coaching, significantly improving self-management outcomes beyond any single technology. Similar synergies are evident in COPD (wearable respiratory monitoring + AI exacerbation prediction + voice follow-up) and multimorbidity care. This integrated framework shifts chronic disease management from siloed, reactive care to a proactive, closed-loop system.

The integration of emerging information technologies in chronic disease management is evolving rapidly, fueled by global digital health advancements. Key trends include AI maturation, widespread remote monitoring adoption, and shift toward value-based care models.

Over the next 3–5 years, critical research directions are likely to include:

- Multimodal data fusion: combining data from wearables, electronic health records, imaging, and genomics to create comprehensive patient profiles, facilitating early detection and predictive analytics in multimorbidity scenarios.
- Personalized intervention algorithms: developing advanced AI/ML models (including large multimodal language models) for dynamic, individually tailored therapies, with strong emphasis on explainable AI to foster clinical confidence.
- Cost-effectiveness evaluation: conducting rigorous health economic studies of integrated platforms and DTx to inform reimbursement policies and value-based pricing.
- Real-world effectiveness verification: large-scale studies in diverse, unselected populations to confirm efficacy beyond controlled trials, while addressing equity, bias, and scalability in resource-limited settings.

In line with the WHO Global Strategy on Digital Health 2020–2025, these directions hold promise for equitable, proactive chronic disease management, transitioning from reactive to preventive and participatory care paradigms. Interdisciplinary collaboration and robust ethical governance will be vital to achieving this vision.
